# *Bacillus thuringiensis* Cells Selectively Captured by Phages and Identified by Surface Enhanced Raman Spectroscopy Technique

**DOI:** 10.3390/mi12020100

**Published:** 2021-01-20

**Authors:** Salvatore Almaviva, Antonio Palucci, Eleonora Aruffo, Alessandro Rufoloni, Antonia Lai

**Affiliations:** 1 ENEA, Italian Agency for New Technologies Energy and Sustainable Development, Via Enrico Fermi 45, I-00044 Frascati, Italy; antonio.palucci@enea.it (A.P.); eleonora.aruffo@unich.it (E.A.); alessandro.rufoloni@enea.it (A.R.); antonia.lai@enea.it (A.L.); 2 Department of Psychological, Health and Territorial Sciences, University “G. d’Annunzio,” Via dei Vestini, 31, I-66100 Chieti, Italy

**Keywords:** SERS, Raman, biosensors, *Bacillus thuringiensis*, *Bacillus anthracis*, BtCS33 phages

## Abstract

In this work, the results on the detection and identification of *Bacillus thuringiensis* (*Bt*) cells by using surface-enhanced Raman spectroscopy (SERS) are presented. *Bt* has been chosen as a harmless surrogate of the pathogen *Bacillus anthracis* (*Ba*) responsible for the deadly Anthrax disease, because of their genetic similarities. Drops of 200 μL of *Bt* suspensions, with concentrations 10^2^ CFU/mL, 10^4^ CFU/mL, 10^6^ CFU/mL, were deposited on a SERS chip and sampled after water evaporation. To minimize the contribution to the SERS data given by naturally occurring interferents present in a real scenario, the SERS chip was functionalized with specific phage receptors BtCS33, that bind *Bt* (or *Ba*) cells to the SERS surface and allow to rinse the chip removing unwanted contaminants. Different chemometric approaches were applied to the SERS data to classify spectra from *Bt*-contaminated and uncontaminated areas of the chip: Principal Component Regression (PCR), Partial Least Squares Regression (PLSR) and Data Driven Soft Independent Modeling of Class Analogy (DD-SIMCA). The first two was tested and trained by using data from both contaminated and un-contaminated chips, the last was trained by using data from un-contaminated chips only and tested with all the available data. All of them were able to correctly classify the SERS spectra with great accuracy, the last being suitable for an automated recognition procedure.

## 1. Introduction

Nowadays the rapid detection and identification of microorganisms in the environment, including aerosol, water and food, is a major issue to prevent large numbers of infections [1] for public health, industrial processes and in the case of a bio-terroristic attack.

For this purpose, fast analytical techniques, not requiring time-demanding samples preparation and suitable to be applied in a continuously running device, are strongly desired.

In this context Surface-Enhanced Raman Spectroscopy (SERS) [2] has been successfully used as a label-free bio-sensing optical technique for rapid identification of bacteria and related spores [3], thanks to its ability to identify molecules from their intrinsic vibrational modes [2,3]. In SERS the inherently weak Raman signal is strongly enhanced, because of the proximity of the sampled molecules to a nanostructured noble metal surface (generally gold or silver but also some non-metallic materials) [4] and this enhancement has been employed for the detection and identification of bacterial pathogens by several groups [5,6,7,8,9] reaching detection limits down to single cell [9].

The SERS enhancement comprises two multiplicative contributions: the electromagnetic enhancement (Em_SERS_) and the chemical one (Chem_SERS_), whose features are briefly summarized in the following.

Em_SERS_ originates from the excitation of surface plasmons at the surface of the SERS substrate, when illuminated with laser light. These surface plasmons induce an enhanced e.m. field, which is experienced by the molecules residing on the surface and is much stronger than the field they would experience without the enhancing substrate. Em_SERS_ is a feature typical of the substrate, independent of the type of molecule and is the strongest contribution to the SERS enhancement, reaching values up to 10^8^–10^10^ [10]. In order to be effective, it requires the molecule to be placed not too far from the substrate (about 1 to 10 nm away from the surface) [10]. For this reason, it is considered a long-range effect when compared to the length of a chemical bond.

Chem_SERS_ originates from the adsorption of the target molecule on the SERS substrate, for which a strong perturbation of the molecule’s structure could arise and chemical bonds between the molecule and the substrate could form. Hence the electronic and geometrical structure of the molecule is altered and the interaction between the molecule and the substrate promotes the creation of substrate–molecule charge transfer states. If the laser source is in resonance or pre-resonance with these states, some Raman modes can be strongly enhanced, in particular those ones coupled to the allowed resonant Raman transitions [11]. Chem_SERS_ can reach values up to 10^2^–10^4^ and requires contact or very small separation (few Angstroms) [12] between the molecule and the substrate, so it is a short-range effect (comparable to the length of a chemical bond). Anyway, Chem_SERS_ plays an important role because it determines the spectral pattern of the SERS spectrum (i.e., the Raman bands position in the spectrum and their intensity ratios) which could significantly differ from the standard Raman pattern of the same substance [11,12].

SERS is particularly suitable for a real time detection of bio-hazards because the typical time required to acquire a spectrum is of the order of seconds or minutes [2,3,4]. For the above-mentioned properties, SERS has been proposed as pre-alarm sensing technique in the framework of the European Defense Agency project RAMBO [13] (Rapid-Air Monitoring particles against BiOlogical threats).

RAMBO aimed at developing advanced methods, instrumentation and sensing strategies/protocols for continuous monitoring of air particles against biological threats, in particular *Ba*, with the following objectives:

(1) good selectivity (low probability of False Alarms);
(2) detect-to-warn response time within 60 min;
(3) minimal use of chemicals and reagents (for long-term unattended operation);
(4) state of the art sensitivity (towards one unit limit);
(5) man-portability with a ruggedized design for use in defense and security scenarios
(6) suitability for a broader biological set of samples, that is, extendable to other microorganisms and toxins.

The underlying idea is that a rapid detection of the threat allows to activate precautionary measures, including prompt prophylaxis against *Ba*, which is classified as a biosafety level 3 bioagent and responsible for Anthrax, a severe, fortunately rare, acute infection involving the skin, the gastrointestinal tract and the lungs [14].

To reach these objectives, two sensing techniques are employed in sequence in RAMBO: SERS and Polymerase-Chain-Reaction analysis (PCRa) [15,16,17]. When SERS, operating as a pre-alarm technique with high sensitivity and fast response time (within 10–15 min), detects a possible alarm, PCRa performs an unambiguous, genetic classification/identification of the bio-agent, with high selectivity and response time within 45 min [18,19]. In this configuration, PCRa does not operate continuously with long duty cycles between subsequent analyses but it is activated only if there is a possible bio-hazard. To reduce the occurrence of false alarms, the SERS sensor is functionalized with specific Gamma-phages, a type of virus that infects bacteria as receptor. These Gamma-phage, selectively captures bacteria of the *Bacillus cereus* group, of which *Ba* is member. Indeed, in addition to *Ba*, the *Bacillus cereus* group includes six very closely related species: *B. cereus* (in strict sense), *B. thuringiensis*, *B. mycoides*, *B. pseudomycoides and B. weihenstephanensis* [20]. In particular, *B. thuringiensis*, (*Bt*) exhibits such a high genetic affinity to *Ba* [20,21,22,23] that it was chosen as its simulant because it is a Gram-positive bacterium harmless to humans and vertebrates [24].

This work presents the results of *Bt* cells detection at very low concentration, down to the detection of a single cell by using SERS as a pre-alarm technique. According to the RAMBO specifications, the detection was carried out in few minutes, over a relatively large area of the sensor, to obtain more reproducible SERS spectra and to reduce the signal variability.

## 2. Materials and Methods

### 2.1. Bacteria Samples and Deposition on SERS Substrates

*Bt* (ATCC^®^ 10792TM) cells were provided by the Military Institute of Hygiene and Epidemiology of Warsaw (Poland), as 1 mL suspensions of vegetative cells in physiological sterile water (0.9% NaCl) with concentration 10^9^ CFU/mL. The samples were then diluted with pure sterile water (MilliQ, Millipore) at concentrations down to 10^2^, 10^4^, 10^6^ CFU/mL. A drop of each suspension, 200 μL vol., was then deposited on a SERS active substrate Klarite [25] and dried. Each drop formed an area of about 200 μm diameter on Klarite for an average density of 6.4·10^−4^, 6.4·10^−2^, 6.4 cells/μm^2^. After deposition and drying of the drop, the cells were deeply rinsed with pure sterile water, to remove residual NaCl and cells not anchored on the SERS surface.

### 2.2. SERS Substrates and Functionalization

Klarite substrates consist of regular arrays of inverted pyramidal pits, realized depositing a sputtered gold layer on a silicon substrate with an ordered nanostructure produced by electron beam lithography. The full chip active area is 4 mm × 4 mm. Each pit has an aperture of 1.5 μm × 1.5 μm and a total pitch size (aperture plus distance to the next microcavity) of 2 μm × 2 μm with a fixed apex pit angle of 70.5°. The cavity depth is 1.06 μm. The regular arrangement of the nanostructures favors a uniform enhancement of the weak Raman signal over the whole excited area, providing a uniform distribution of “hotspots” [4] and a more reproducible SERS signal.

**Figure 1:**
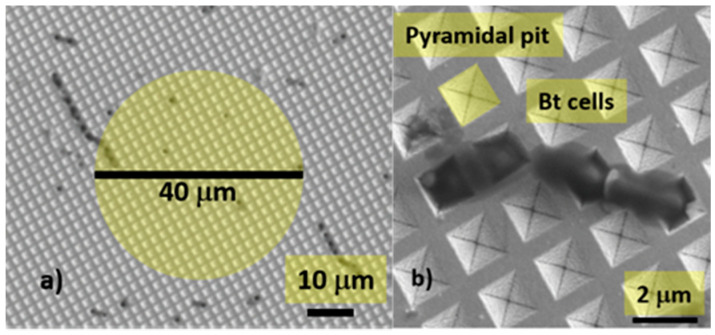
(**a**) Scanning Electron Microscope (SEM) image of a *Bt*-contaminated surface-enhanced Raman spectroscopy (SERS) substrate. The yellow circle shows the dimension of the laser spot and, consequently, the scanned area (**b**) some *Bt* cells deposited on the substrate, a pyramidal pit is highlighted in yellow.

Figure 1 shows two Scanning Electron Microscope (SEM) images of a Klarite substrate at different magnifications, with a pyramidal pit, highlighted in yellow, forming the SERS active “hot spot” and some *Bt* cells deposited on the substrate.

The substrates were functionalized with *Bt*CS33 phages [26,27] at the Institut de Chimie et Biochimie Moléculaires et Supra-moléculaires of the Université Lyon1-CNRS (France) according to the following protocol:

1. SERS surfaces were first coated using carboxy-aniline electrodeposition through cyclic voltammetry (3 cycles, from 0 to −2 V vs. Pt at a scan rate of 200 mV/sec).
2. The grafted carboxylic acid function was activated using carbodiimide (EDC 0.4M + NHS 0.05M for 8 min at room temperature).
3. The phages were then brought in contact with the activated surface (phages over-concentrated in acetate buffer 0.1 M, pH 6 for 16 min at room temperature).
4. The activated surface was then neutralized using 1 M ethanolamine, pH 8.5.

Between each step, the SERS surface was washed with MilliQ sterile water (18 MΩ), air dried and stored under vacuum at 4 °C.

### 2.3. SERS Measurements

SERS measurements were performed with a table-top micro-Raman system “*i*-Raman” by Bw&Tek, equipped with a GaAlAs 785 nm diode laser (linewidth < 0.3 nm). The spectral range spans between 100–3500 cm^−1^ and the spectral resolution is 3.5 cm^−1^. The laser power was set to 60 mW, focused through a 40× objective, 0.5 N.A. in a spot of 40 μm diameter (Figure 1a). The energy density in (for) the scanned area is then 1.43 mJ/μm^2^. The relatively large scanned area limits the fluctuations of the SERS signal in different points of the sample, resulting in a more reproducible signal. Taking into account the drop diameter (200 μm) and the laser spot diameter (40 μm) the average number of sampled cells for each dilution was between 0.8 and 8000. The SERS signal from the substrate was dispersed through a 600 lines/mm grating and recorded by a cooled (10 °C) Charge Couple Device (CCD) array detector (2048 pixels, 16 digital bits). Each recorded spectrum was the average of 3 subsequent acquisitions of 10 s integration time, for a total integration time of 30 s.

## 3. Results

### SERS Spectra of Bt Cells on Functionalized Substrate

The collected SERS spectra were processed by applying the following steps (1) cropping of the spectral region between 350–1800 cm^−1^; (2) fluorescence background subtraction; (3) spikes removal and (4) normalization (between 0 and 1) of the residual signal. All these steps were performed processing the raw spectra by using home-developed routines, in a Matlab™ environment. In particular, the fluorescence background subtraction was performed with an iterative procedure, similar to the one reported in [28], replacing the standard high-order polynomial function with a new fitting function obtained from an iterative data processing algorithm. Briefly, for each iteration the algorithm applies a smoothing process to the original spectrum, obtaining a smoothed version of the latter with reduced local maxima and minima. Then, the algorithm creates a new artificial spectrum, holding both the local maxima of the smoothed spectrum and the local minima of the original spectrum, ready to be processed in further iterations. The final result is a featureless and smoothed curve, anchored to the original minima, without the spectral features corresponding to the local maxima, consistently flattened and suitable to be subtracted to the original spectrum as fluorescence background.

**Figure 2:**
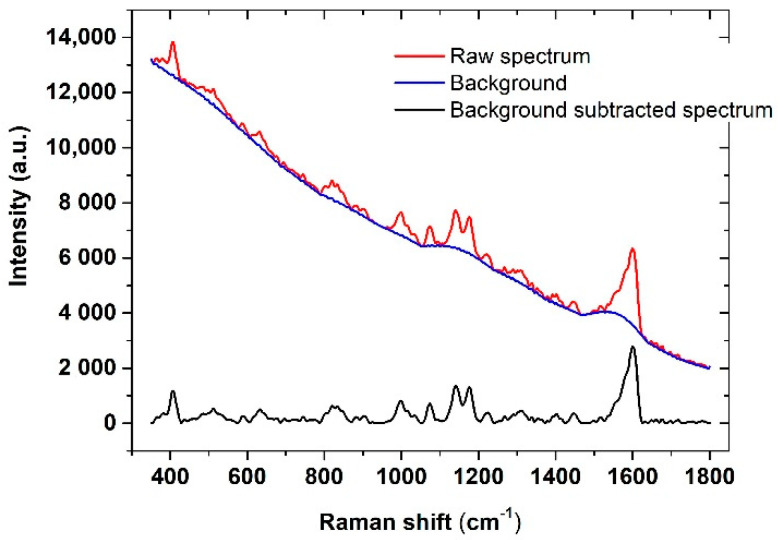
SERS spectrum of the functionalized substrate: Raw spectrum with fluorescence background (red line), fluorescence background (blue line), background-subtracted spectrum (black line).

An example of this procedure, applied to a raw (as-recorded) spectrum, is shown in Figure 2.

A total of 400 spectra were acquired to apply the discrimination/classification procedures: 100 were relative to the uncontaminated substrate; 100 sampled on *Bt*-contaminated substrate with 10^2^ CFU/mL; 100 sampled at 10^4^ CFU/mL concentration and, finally, the remaining 100 at 10^6^ CFU/mL. Spectra were collected performing 5 repetitions on 20 different points and processed by applying the described background subtraction procedure.

**Figure 3:**
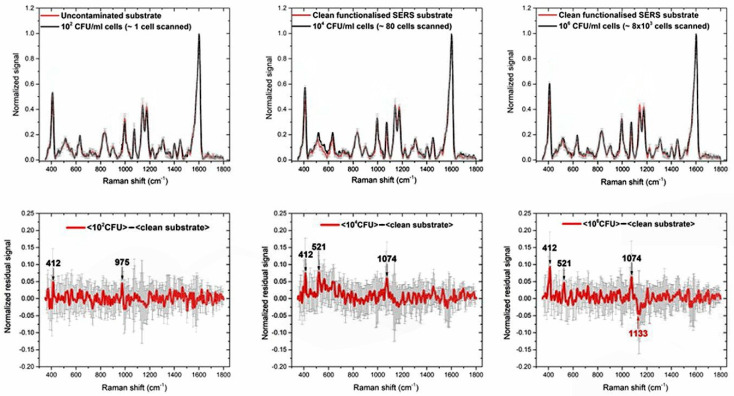
(**Top**) Mean SERS spectra of the contaminated substrate (black lines) with (from left to right) 10^2^ CFU/mL, 10^4^ CFU/mL, 10^6^ CFU/mL *Bt* cells. The red spectrum in each graph is the mean SERS spectrum of the functionalized substrate before inoculation with *Bt* (control, clean substrate). (**Bottom**) Residual signals obtained by subtracting the spectrum of the contaminated substrates (black lines) from the one of the clean substrate (red lines). The more remarkable spectral differences are labelled in black, if the residual signal is positive or red, if negative. Error bars are reported as grey vertical bars in each graph.

Figure 3 (top panels) shows the normalized mean spectra for each concentration (black lines), with relative standard deviations (SD, grey vertical bars), compared with the mean spectrum before inoculation with *Bt* (control, red lines). The high reproducibility of the SERS signal is demonstrated by the small values of the SD in each pixel, which had a mean value of 2–4% considering all the pixels and did not exceed 12.2% in any case.

The evident spectral features at about 412, 521, 632, 834, 900, 997, 1074, 1133, 1176, 1222, 1277, 1307, 1402, 1447, 1603 cm^−1^ are to be ascribed to the overall functionalization process described in Section 2.2: in fact, they are also observed in the clean, un-contaminated substrate. Because of the complexity of the whole process their interpretation in terms of specific Raman bands is not unique. Anyway, this signal represents only the background upon which the signal due to *Bt* cells is detected and the last is inferred in the following as intensity fluctuations of the above-mentioned spectral structures.

The weakness of the signals ascribable to *Bt* cells is explained by the very superficial nature of the SERS enhancement, which is typically relevant only in a layer of few nanometers close to the surface. Then, considering that the functionalization process could result in an interfering layer increasing the distance between the cells and the chip surface, a reduced enhancement of the cells signal is expected [10,11]. To highlight this signal, we subtracted the averaged SERS spectra of the clean substrate from the contaminated ones, looking for the most evident differences. The results are shown in the bottom panels of Figure 3, whereas fluctuations larger than 5% of the highest peak at 1603 cm^−1^, are marked in black if positive and in red if negative. In the first case, they are supposed to represent a residual *Bt* signal, in the second case the presence of *Bt* is supposed to reduce the background signal. Error bars (in grey) are the sum of the SDs of the mean spectra. The experimental results here described highlight the sensitivity of the technique, capable to detect very few cells. However, the problem of label-free detection of large cell structures by SERS methods is well known. It is accepted that, from these measures, the selectivity, i.e., the possibility of discriminating between different types of bacilli, would be guaranteed only by the specific functionalization by *Bt*CS33 phages, designed to bind exclusively *Bt*. For this reason, further tests with different types of bacilli of the Cereus group are needed and planned. However, SERS is considered in RAMBO as an early warning technique, with the task of providing high sensitivity and adequate selectivity, while the genetic identification of the bound organic material is entrusted to the PCRa technique.

## 4. Discussion

### 4.1. Chemometric Analysis of the SERS Spectra: Principal Component Regression and Partial Least Squares Regression Classification Methods

Although the SERS signal of the *Bt* cells appeared weak, to verify the possibility to correctly discriminate between spectra from *Bt*-contaminated or un-contaminated SERS substrates, two different chemometric approaches were applied: Principal Component Regression (PCR) [28,29] and Partial Least Squares Regression (PLSR) [30]. PCR involves Principal Components Analysis (PCA) [31,32,33] to explain and maximize the observed variability in a representative group of data, forming the so-called “training set” that is composed of samples (spectra) from different classes, building a predictive model by using orthogonal, uncorrelated variables called Principal Components (PCs) [28,32]. PLSR also computes PCs running on a training set but builds its model taking preliminarily into account particular “prediction” variables, whose values are typically related to some properties of the samples. Relatively to the measurements under analysis in this work, the prediction variables were set “0” for the spectra of the uncontaminated areas (negative entries) and “1” for those of the *Bt*-contaminated areas (positive entries).

Both PCR and PLSR were trained with training sets of 10, 20, 50, 80, 100 randomly chosen spectra, of which one half were composed of negative entries, the remaining half of positive entries (from the 10^6^ CFU/mL group). Once built, the PCR and PLS models were projected over the whole dataset, assigning a value to the prediction variable for each spectrum. Then, the spectra were classified as negative entries if their prediction variable were closer to zero than one, positive entries otherwise. The classification performances of PCR and PLSR were tested in term of sensitivity (Se) and specificity (Sp) [33,34,35,36,37], defined respectively as the “true positive rate” (i.e., the probability to correctly classify spectra from the contaminated SERS substrate) and “true negatives rate”(i.e., the probability to correctly classify spectra from an uncontaminated one). Se and Sp were computed according to the following relations:

Se = TP/(TP + FN) × 100(1)

Sp = TN/(TN + FP) × 100,(2)

where TP are “true positives,” that is, positive entries correctly classified, TN “true negatives,” that is, negative entries correctly classified, FP are “false positives,” that is, negative entries erroneously classified and FN are “false negatives,” that is, positive entries erroneously classified. To obtain results independent from the particular spectra composing the training set, each training procedure was reiterated 100 times: at each iteration a new training set was composed of randomly chosen spectra acquired by two substrates (uncontaminated and contaminated) and the mean and standard deviation values of TN, TP, FN, FP, Se, Sp were finally computed over these iterations. Table 1 listed the obtained results for both PLSR and PCR: TN, TP, FN, FP, Se, Sp are reported as a function of the number of spectra in the training sets.

It can be observed that for PLSR both Se and Sp tend to increase with the number of spectra, while for PCR, an increasing trend is observed only for Se, whereas an opposite behavior is observed for Sp, suggesting that with larger training sets, PCR tends to build predictive models that erroneously classify negative entries. This can be confirmed also looking at the trends of TN and FP in Table 1. Moreover, it can be also observed that, for smaller training sets, PCR exhibits better performances than PLSR. This could be explained considering that PCR maximizes the variance without taking into account predictors values, as PLSR does, producing misleading results in the case of training sets of few samples. This is also confirmed by observing that PLSR gives better prediction results respect to PCR in the case of larger training sets and probably would exhibit better discrimination capabilities in classifying entries from more classes, for which the prediction variables are more meaningful.

In conclusion, both PCR and PLSR demonstrated the possibility to classify these groups of SERS spectra with high sensitivity and selectivity.

### 4.2. One Classifier Method: DD-SIMCA

**Figure 4:**
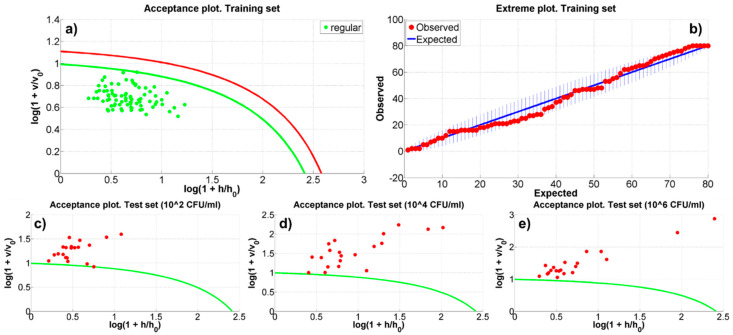
The Data Driven Soft Independent Modeling of Class Analogy (DD-SIMCA) model results relative to the run number 9. Panel (**a**) shows the acceptance plot for the training set. Panel (**b**) shows the extreme plot for the training set (the number of observed extreme objects is within the tolerance area, the blue vertical lines). Panel (**c**–**e**) illustrate the acceptance plots for the BT-contaminated samples with 10^2^, 10^4^ and 10^6^ CFU/mL. In panels (**a**,**c**–**e**), h and v represents the score and orthogonal distance and h_0_ and v_0_ the mean score and orthogonal distance of the training set, respectively.

A further step of automatic recognition and classification could be the implementation of a recognition procedure by using a one-class classifier method (OCC), where only a single group of data, for example the spectra from the uncontaminated samples, is needed. The advantage of an OCC technique is that the training procedure could be preliminarily performed on each new, uncontaminated SERS chip, making it ready to automatically recognize outliers, once the sensor is contaminated and used in a real scenario. For this purpose the Data Driven Soft Independent Modeling of Class Analogy (DD-SIMCA) [37,38] was applied as fast, reliable OCC. To be trained DD-SIMCA requires the definition of a training set of data from a target class (i.e., the spectra of the uncontaminated substrate). The method applies the PCA to this dataset and represents each member as a point in a 2D-space, where the score distance (hi) and the orthogonal distance (νi), calculated by the PCA, are the coordinates. Then, for a given parameter (the α-value), the DD-SIMCA defines in this 2D-space an acceptance area (or threshold) within which to include all the elements of the target class (panel (a) in Figure 4). It is, then, possible to distinguish between points (data) belonging to the target class and aliens, that is, data that are located outside the acceptance area and classifiable as non-members of the target class (panels (c–e) in Figure 4). Moreover, an outlier’s analysis can be carried out introducing a further cut-off curve in the acceptance plot. An extreme plot allows to verify the quality of the classification for a given number of PCs, showing the relation between the observed and the expected number of extreme samples (panel (b) in Figure 4).

To perform DD-SIMCA on the acquired SERS data a Matlab™ tool developed by Zontov et al. [39] has been used. In the present analysis, the 80% of the uncontaminated samples has been randomly selected to produce the training set and, consequently, to define the model. The 20% of each (i.e., 20 spectra) of the remaining three datasets has been randomly extrapolated to define the three test sets that have to be classified. The training set has been pre-processed rescaling the data between −0.5 and 0.5. The chosen setup of the model parameters is: (a) 15 and 20 PCs have been considered; (b) α-value has been automatically evaluated by the DD-SIMCA tool; (c) the outliers significance has been fixed to 0.01, (d) the acceptance area has been defined as chi-square type (i.e., a triangular area calculated as the sum of the normalized score and orthogonal distances) and (e) the classic method has been used to estimate the chi-square distribution parameters. This model setting allowed to ensure that the number of observed extreme objects in the training dataset is included in the tolerance area (as shown in panel (b) of Figure 4). The model has been run for 10 different randomly generated datasets of training and test samples. Table S1 reports the results of the DD-SIMCA model and Figure 4 shows, as an example, the acceptance plot for both the training and the test datasets (spectra from the contaminated substrates) and the extreme plot relative to the ninth run. In synthesis, DD-SIMCA did not classify any aliens or outliers within the 80 spectra that defined the training sets. On the contrary, both the configurations with 15 and 20 PCs, generally, classified the spectra of the contaminated substrates as outliers. In details, the 15 PCs models in 7 cases (i.e., ~7.8%, yellow boxes in Table S1 identified test samples as part of the target classes 1 or 2. Finally, the 20 PCs models showed outliers in the extreme plots in 4 runs.

## 5. Conclusions

In this paper, the ability of SERS spectroscopy to detect low numbers of *Bt* cells was presented and discussed based on the discrimination capabilities of the chemometric techniques PCR, PLSR and DD-SIMCA. SERS spectra acquired from SERS functionalized substrates contaminated with *Bt* cells (simulating the deadly Bacillus anthracis) were collected and compared with those acquired from an uncontaminated one, being correctly classified as potentially dangerous with high accuracy. At the lower *Bt* concentration of 10^2^ CFU/mL, the collected spectra accounted, on average, for only one *Bt* cell on the scanned area. PCR and PLSR required no more than 100 sample spectra to be preliminarily trained, reaching selectivity and specificity percentages close to 100% on test sets. DD-SIMCA was tested as an OCC method suitable to automatically recognize possible threats in a realistic scenario. The obtained results show that SERS spectroscopy has the potential to detect biological pathogens in real time, with minimal sample preparation and with high sensitivity down to single cell and is eligible as a pre-alarm, fast, preliminary diagnostic technique, that could be followed by subsequent unambiguous, genetic-based classification/identification techniques like PCRa in the case of the RAMBO project.

## Figures and Tables

**Table 1:** Classification results for Partial Least Squares Regression (PLSR) and Principal Component Regression (PCR) for different test sets. In the yellow boxes are reported the resulting Se and Sp as calculated from Equations (1) and (2).

# Spectra in the Training Set		10	20	50	80	100
PLSR	TN	85 ± 1	84 ± 2	90 ± 1	90 ± 2	92 ± 2
	TP	209 ± 26	251 ± 27	290 ± 9	292 ± 8	295 ± 6
	FN	91 ± 26	49 ± 27	10 ± 9	8 ± 8	5 ± 6
	FP	15 ± 1	16 ± 2	10 ± 1	10 ± 2	8 ± 2
	Se (%)	85 ± 25	84 ± 24	90 ± 8	90 ± 7	92 ± 6
	Sp (%)	70 ± 2	84 ± 5	97 ± 3	97 ± 6	98 ± 6
PCR	TN	89 ± 0	89 ± 0	91 ± 0	86 ± 5	80 ± 7
	TP	294 ± 0	294 ± 0	293 ± 0	294 ± 2	296 ± 2
	FN	6 ± 0	6 ± 0	7 ± 0	6 ± 2	4 ± 2
	FP	11 ± 0	11 ± 0	9 ± 0	14 ± 5	20 ± 7
	Se (%)	89 ± 0	89 ± 0	91 ± 0	98 ± 2	99 ± 2
	Sp (%)	98 ± 0	98 ± 0	98 ± 0	86 ± 15	80 ± 22

## Supplementary Materials

The following are available online at https://www.mdpi.com/2072-666X/12/2/100/s1, Table S1: The DD-SIMCA model results. The yellow boxes represent the simulations in which the model did not identify as extraneous respect to the current model all the test samples.
