# Supplementary material for: Bacillus thuringiensis Cells Selectively Captured by Phages and Identified by Surface Enhanced Raman Spectroscopy Technique
Source: Micromachines (Basel). 2021 Jan 20;12(2):100. doi: 10.3390/mi12020100 (PMC7909556; doi:10.3390/mi12020100)
Supplement: Supplementary file 1 [file micromachines-12-00100-s001.pdf]

Run	Training results	15 PCs	20 PCs	Test samples	Test results	15 PCs	20 PCs
**1**	α	0.0009	0.0057	10^2 CFU/ml	β	0.1308	-
	Samples	80	80		Samples	20	-
	Extremes	0	0		Externals	18	-
	Outliers	0	0	10^4 CFU/ml	β	0.1364	-
	Notes		*The extreme plot shows outliers.*		Samples	20	-
					Externals	18	-
				10^6 CFU/ml	β	0.010	-
					Samples	20	-
					Externals	20	-
**2**	α	0.015	0.024	10^2CFU/ml	β	0.0691	5.2699e-05
	Samples	80	80		Samples	20	20
	Extremes	0	0		Externals	20	20
	Outliers	0	0	10^4 CFU/ml	β	0.003	7.6536e-05
	Notes		*The extreme plot shows a couple of outliers.*		Samples	20	20
					Externals	20	20
				10^6 CFU/ml	β	0.0002	3.951e-07
					Samples	20	20
					Externals	20	20
**3**	α	0.0063	0.0296	10^2CFU/ml	β	0.0214	0.0008
	Samples	80	80		Samples	20	20
	Extremes	0	0		Externals	20	20
	Outliers	0	0	10^4 CFU/ml	β	0.0005	1.1640e-05
	Notes				Samples	20	20
					Externals	20	20
				10^6 CFU/ml	β	0.0007	7.5937e-06
					Samples	20	20
					Externals	20	20
**4**	α	0.0153	0.0221	10^2CFU/ml	β	0.0872	0.0061
	Samples	80	80		Samples	20	20
	Extremes	0	0		Externals	20	20
	Outliers	0	0	10^4 CFU/ml	β	0.0505	0.0032
	Notes				Samples	20	20
					Externals	20	20
				10^6 CFU/ml	β	0.001	9.2492e-07
					Samples	20	20
					Externals	20	20
**5**	α	0.0051	0.0166	10^2CFU/ml	β	0.0117	2.1159e-05
	Samples	80	80		Samples	20	20
	Extremes	0	0		Externals	19	20
	Outliers	0	0	10^4 CFU/ml	β	0.0056	5.9069e-05
	Notes				Samples	20	20
					Externals	20	20
				10^6 CFU/ml	β	0.9084	0
					Samples	20	20
					Externals	18	20
**6**	α	0.0232	0.0270	10^2CFU/ml	β	0.06199	-
	Samples	80	80		Samples	20	-
	Extremes	0	0		Externals	19	-
	Outliers	0	0	10^4 CFU/ml	β	8.4298e-05	-
	Notes		*The extreme plot shows outliers.*		Samples	20	-
					Externals	20	-
				10^6 CFU/ml	β	2.5971e-05	-
					Samples	20	-
					Externals	20	-
**7**	α	0.0048	0.0045	10^2CFU/ml	β	0.1283	0.0119
	Samples	80	80		Samples	20	20
	Extremes	0	0		Externals	20	20
	Outliers	0	0	10^4 CFU/ml	β	NaN	NaN
	Notes		*The extreme plot shows a couple of outliers.*		Samples	20	20
					Externals	20	20
				10^6 CFU/ml	β	0.0021	1.2783e-05
					Samples	20	20
					Externals	20	20
**8**	α	0.0018	0.0155	10^2CFU/ml	β	0.1117	0.0005
	Samples	20	20		Samples	20	20
	Extremes	0	0		Externals	19	20
	Outliers	0	0	10^4 CFU/ml	β	0.2548	0.0015
	Notes				Samples	20	20
					Externals	19	20
				10^6 CFU/ml	β	0.0013	2.7799e-06

					Samples	20	20
					Externals	20	20
**9**	α	0.0078	0.0018	10^2CFU/ml	β	0.0777	0.0051
	Samples	80	80		Samples	20	20
	Extremes	0	0		Externals	20	20
	Outliers	0	0	10^4 CFU/ml	β	0.0594	0.0015
	Notes		*The extreme plot shows a couple of outliers.*		Samples	20	20
					Externals	20	20
				10^6 CFU/ml	β	0.0058	0.0001
					Samples	20	20
					Externals	20	20
**10**	α	0.0185	0.0104	10^2CFU/ml	β	0.0279	0.0029
	Samples	80	80		Samples	20	20
	Extremes	0	0		Externals	20	20
	Outliers	0	0	10^4 CFU/ml	β	0	0
	Notes				Samples	20	20
					Externals	20	20
				10^6 CFU/ml	β	5.4578	0
					Samples	20	20
					Externals	20	20

Table S1. The DD-SIMCA model results. The yellow boxes represent the simulations in which the model did not identify as extraneous respect to the current model all the test samples.
